# QuickVue Influenza A+B rapid test for influenza surveillance in community

**Published:** 2011-01-10

**Authors:** Calvin KY Cheng, Dennis KM Yip, Kwok Hung Chan, JS Malik Peiris, Benjamin J Cowling

**Affiliations:** 1 Department of Community Medicine and School of Public Health, The University of Hong Kong, Hong Kong SAR; 2 Department of Microbiology, Queen Mary Hospital, Hospital Authority, Hong Kong, Hong Kong SAR; 3 HKU Pasteur Research Centre, Hong Kong, Hong Kong SAR

## Background

Rapid diagnosis of influenza not only facilitates timely clinical management, time series test results can also serve for disease surveillance purpose. We evaluated the performance of a community based system using QuickVue Influenza A+B test (Quidel Corp., San Diego, California) for influenza surveillance in Hong Kong.

## Methods

As part of a large community study, subjects older than 2 years reporting at least two symptoms of influenza-like-illness were recruited from 30 outpatient clinics in Hong Kong prospectively from February 2007 to July 2010 around the influenza seasons [1,2]. Each subject provided a pooled pair of nose and throat swabs to be tested by the QuickVue rapid test on site. Overall test positive rate were weekly aggregated and compared with a hospital laboratory surveillance system (Weekly influenza virus isolation rate from the Queen Mary Hospital, Hong Kong) and a pre-existing community influenza sentinel surveillance system (Weekly consultation rate of influenza-like illness reported by general practitioners in private practice from the Centre of Health Protection, Department of Health, HKSAR).

## Results

A total of 5,824 subjects were recruited throughout the study. We define each study week as either high influenza activity or low influenza activity using fixed thresholds after removing extreme values two standard deviations above the mean. The sensitivity of QuickVue data for picking up high influenza activity was 78% when compare with the laboratory data and was 100% when comparing with the community data.

## Conclusion

Although the sensitivity of QuickVue Influenza A+B test decreases with specimen viral load, in which may miss out mild influenza infection cases [3], its excellent specificity allows more accurate influenza surveillance when compare with other syndromic based systems. Its point-of-care usage and easy-to-use nature also increase the flexibility and popularity for disease surveillance, especially in resource limited setttings where laboratory facilities and expertise are not available.
